# Proposal of standardization of every step of angiographic procedure in bleeding patients from pelvic trauma

**DOI:** 10.1186/s40001-021-00594-8

**Published:** 2021-10-14

**Authors:** Matteo Renzulli, Anna Maria Ierardi, Nicolò Brandi, Sofia Battisti, Emanuela Giampalma, Giovanni Marasco, Daniele Spinelli, Tiziana Principi, Fausto Catena, Mansoor Khan, Salomone Di Saverio, Giampaolo Carrafiello, Rita Golfieri

**Affiliations:** 1 Department of Radiology, IRCCS Azienda Ospedaliero-Universitaria di Bologna, Via Albertoni 15, Bologna, Italy; 2 Radiology Unit, Department of Experimental, Diagnostic and Specialized Medicine, Sant’Orsola Hospital, University of Bologna, Bologna, Italy; 3 Radiology Department, Fondazione IRCCS Cà Granda Ospedale Maggiore Policlinico, Milan, Italy; 4 Radiology Unit, Bufalini Hospital, Cesena, Italy; 5 Division of Internal Medicine, IRCCS Azienda Ospedaliero-Universitaria di Bologna, Bologna, Italia; 6 Intensive Care Unit and Anesthesia, Emergency Department, ASUR MARCHE AV5, San Benedetto del Tronto, Italy; 7 Department of Emergency and Trauma Surgery, Parma University Hospital, Parma, Italy; 8 Digestive Diseases Department, Brighton and Sussex University Hospitals, Brighton, UK; 9 Royal College of Surgeons of England, DSTS Faculty, London, UK; 10 Department of General Surgery, University of Insubria, University Hospital of Varese, ASST Sette Laghi, Varese, Regione Lombardia Italy

**Keywords:** Pelvic trauma, Angioembolization, Angiography, Bleeding, Interventional radiology

## Abstract

Trauma accounts for a third of the deaths in Western countries, exceeded only by cardiovascular disease and cancer. The high risk of massive bleeding, which depends not only on the type of fractures, but also on the severity of any associated parenchymal injuries, makes pelvic fractures one of the most life-threatening skeletal injuries, with a high mortality rate. Therefore, pelvic trauma represents an important condition to correctly and early recognize, manage, and treat. For this reason, a multidisciplinary approach involving trauma surgeons, orthopedic surgeons, emergency room physicians and interventional radiologists is needed to promptly manage the resuscitation of pelvic trauma patients and ensure the best outcomes, both in terms of time and costs. Over the years, the role of interventional radiology in the management of patient bleeding due to pelvic trauma has been increasing. However, the current guidelines on the management of these patients do not adequately reflect or address the varied nature of injuries faced by the interventional radiologist. In fact, in the therapeutic algorithm of these patients, after the word “ANGIO”, there are no reports on the different possibilities that an interventional radiologist has to face during the procedure. Furthermore, variations exist in the techniques and materials for performing angioembolization in bleeding patients with pelvic trauma. Due to these differences, the outcomes differ among different published series. This article has the aim to review the recent literature on optimal imaging assessment and management of pelvic trauma, defining the role of the interventional radiologist within the multidisciplinary team, suggesting the introduction of common and unequivocal terminology in every step of the angiographic procedure. Moreover, according to these suggestions, the present paper tries to expand the previously drafted algorithm exploring the role of the interventional radiologist in pelvic trauma, especially given the multidisciplinary setting.

## Introduction

Trauma accounts for a third of deaths in Western countries, exceeded only by cardiovascular disease and cancer. Most of all, trauma represents the most common cause of death and disability under the age of 44 years in the United States, with a growing number of deaths over the years: in fact, the number of deaths has increased by 22.8% in the decade between 2000 and 2010, having reached a peak of 170,000 deaths in 2010 [1]. In Italy, the analysis of the largest epidemiological database of trauma demonstrated almost 15,500 deaths secondary to trauma during 2002 [2]. Since trauma mainly affects the active population and its sequelae can be chronic, potentially causing severe disability and negatively impacting the social sphere and working place, it constitutes a huge economic burden both for the victims and their families [3].

Among all the possible fractures that can occur after a trauma, pelvic fractures are relatively uncommon, occurring mainly in high-speed traffic accidents or falls from height, with a reported incidence of 10% of all fractures [4]. However, their frequency can increase up to 25% in the polytraumatized patient [5]. A prompt diagnosis of pelvic fractures is essential due to the risk of a massive hemorrhage associated with this type of trauma. In fact, the high risk of massive bleeding, which depends not only on the type of fractures, but also on the severity of any associated parenchymal injuries makes pelvic fractures one of the most life-threatening skeletal injuries, with a high mortality rate (ranging from 5–50%) [6–8]. Therefore, pelvic trauma represents an important condition to recognize early, manage, and treat. For this reason, a multidisciplinary approach involving trauma surgeons, orthopedic surgeons, emergency room physicians and interventional radiologists is needed to promptly manage the resuscitation of pelvic trauma patients and ensure the best outcomes, both in terms of time and costs [9]

Pelvic hemorrhages are caused by the destruction of bone structures with subsequent vascular bleeding, caused by venous injuries in the vast majority of cases. However, 3–15% of patients who sustained pelvic fractures demonstrate arterial bleeding [10–13]. Pelvic fracture hemorrhages caused by venous injury at the fracture site can be effectively treated with external fixators, C-clamps, and belts by reducing the pelvic volume and stabilizing the fracture [14]. On the other hand, in cases of arterial bleeding, resuscitation and stabilization of the pelvis are often not enough to stop the bleeding source, and an urgent angiography followed by angioembolization (AE) has been proven to be a safe and effective treatment [15]. In fact, over the last 40 years, AE has revolutionized the management of pelvic trauma, demonstrating to be over 90% effective in controlling hemorrhage in the abdomen and pelvis from both blunt and penetrating trauma [16].

Despite the fact that many studies have been published regarding the role and indications for angiography in pelvic trauma, no standardized angiographic operating procedure has been developed yet on the different steps.

In fact, the angiographic management of patients with bleeding pelvic trauma still remains controversial, with plenty of variations in indications, but also in techniques and protocols [17]. The lack of the necessary strong scientific evidence that leads to univocal methodological management of these patients when they were admitted to the angiographic room is probably due to the limitations of published series concerning the role of AE in pelvic trauma. In fact, AE has been performed with differing indications and techniques, leading to varied outcomes as a consequence of the absence of standardization in every step of the angiographic procedures. Therefore, it is mandatory to standardize angiographic procedures in the setting of pelvic trauma.

This article aims to review the recent literature on optimal imaging assessment and management of pelvic trauma, defining the role of the interventional radiologist within the multidisciplinary team, suggesting the introduction of common and unequivocal terminology in every step of the angiographic procedure. Moreover, starting from a critical analysis of angiographic techniques, this paper proposes a new specific angiographic algorithm in order to achieve an optimal application of AE detailing any angiographic steps in pelvic trauma patients.

## Methodology

We reviewed the available literature on AE for the optimal imaging assessment and management of pelvic trauma to assess indications for the radiological interventional procedure, including relationship to fracture type, CT findings and complications. A comprehensive search was carried out in different databanks (MEDLINE, SCOPUS, PUBMED) for articles in the English language. The terms and keywords used included pelvis, pelvic, injuries, trauma, fractures, external fixation, internal fixation, hemodynamic instability/stability, angioembolization, pelvic binder/binding, aortic, balloon, occlusion, stabilization and computed tomography; no search restrictions were imposed.

## Treatment for hemorrhage in pelvic fracture: state of the art

### Pelvic stabilization

There are multiple options available for controlling the pelvic fracture-related hemorrhage. Since the vast majority of bleeding in pelvic fractures is venous, the pelvic stabilization with a C-clamp (in type C fractures) or the external fixator (in type B fractures) represent the first maneuver for controlling pelvic fracture-related hemorrhage in all patients, decreasing the pelvic volume, permitting tamponade to occur and allowing a clot to stabilize, regardless the hemodynamic status [18, 19]. In hemodynamically unstable patients, preperitoneal pelvic packing (PPP) should be performed along with external fixation, since it provides a counter-pressure from the pelvic ring to the applied lap sponges in the retroperitoneal space [20].

The outcomes and effectiveness of pelvic stabilization and PPP in controlling hemorrhages secondary to pelvic trauma are not homogeneous in the varying series published in the literature. Cothren et al. [21] demonstrated that external fixation and PPP could be enough to control bleeding in severely injured patients with pelvic fractures, with lower mortality compared with historic data: in fact, only 17% of patients in their series, required a subsequent AE for ongoing hemorrhage. However, the same group later published another study, which, after using PPP as the primary treatment for pelvic hemorrhage, 27% of patients still had to undergo angiographic intervention [22]. In Sandu et al. [23] nearly 58% of the patients who underwent PPP showed active arterial bleeding in subsequent angiography.

The reason why external fixation and PPP were unsuccessful in controlling hemorrhage in this large percentage of cases is unclear. One of the possible explanations is that the percentage of bleeding from the arterial source in pelvic fractures is higher than that reported in the vast majority of published series. Moreover, differences in cohort selection, as well as the lack of a unanimous and standardized protocol for the management of pelvic trauma, may be responsible for such different results.

These findings imply that PPP should be used as a bridge measure when the bleeding is not controlled, or when angiography is not immediately available. In these clinical scenarios and circumstances, PPP should be considered a synergic treatment rather than a competing therapy with AE in controlling pelvic fracture-related hemorrhage.

### Resuscitative endovascular balloon occlusion of the aorta (REBOA)

The REBOA technique, as the name suggests, involves the introduction of a balloon catheter via the femoral artery into the aorta. Once inflated, the balloon provides a total occlusion of the aorta either just above the diaphragm (Zone I), to control intra-abdominal bleeding, or above the aortoiliac bifurcation (Zone III), to control bleeding in the pelvis or proximal extremities [24]. The total occlusion of the aorta between the celiac trunk and the lowest renal artery (Zone II) is not utilized [24].

REBOA is a potential life-saving intervention in patients presenting with critically uncontrolled hemorrhagic shock after pelvic fracture, bridging the time to the operating room (OR) until definitive surgical bleeding control [20, 25]. REBOA seems to be superior to standard treatment in very sick patients with impending cardiac arrest or cardiac arrest, as an alternative to resuscitative thoracotomy [26]. However, complications of REBOA are numerous, including aortic dissection, rupture and perforation, embolization, peripheral ischemia, and multiple organ failure related to a long-term occlusion [27]. The evidence for its efficacy is limited and contradictory, as evident by comparing data of patients treated with and without REBOA. In fact, REBOA placement in severely injured trauma patients has been associated with higher rates of mortality, acute kidney injury, and lower extremity amputation compared with similarly injured trauma patients without REBOA in a Nationwide Analysis regarding civilian trauma [28]. Furthermore, Mikdad et al. [29] suggest that early PPP may offer a benefit over REBOA in the setting of hemorrhage after pelvic trauma, improving survival rates.

Another disadvantage of REBOA is that this technique represents only a temporary solution to control active bleeding, waiting for definitive bleeding control. Finally, the described limitations of the REBOA technique may explain why this technique is not used in most centers dedicated to trauma, such as in our institution.

### Angioembolization (AE)

Along with pelvic stabilization, AE has overlapping, complementary, and sometimes, competing role in controlling pelvic fracture-related hemorrhage [30]. The importance of AE derives from the difficulty of stopping the certain forms of arterial bleeding with laparotomy. In fact, even if surgery provides hemostasis through the opening of the retroperitoneum and the isolation of the lacerated vessels, the surgical procedure itself causes the loss of the natural hemostatic effect on the source of bleeding sustained by the hematoma itself and by the retroperitoneal fascia, with an elevated risk of a sudden and significant increase in hemorrhage [31].

The use of the angiographic intervention in pelvic trauma depends on several factors, including the patient’s hemodynamic status and CT findings. Generally, the basic indication for angiography is the suspicion of an injured artery in pelvic trauma. In particular, well-accepted indications for AE are both direct and indirect CT signs of arterial bleeding [32–34]. In fact, it has been reported that contrast extravasation in the arterial phase of contrast-enhanced CT (seen as a contrast “blush”) is a highly predictive direct sign of active arterial bleeding, with sensitivity and specificity values of 82% and 95%, respectively [35, 36]. CT blush closely correlates with the angiographic findings and thus guides the interventional radiologist to selectively study the arteries most likely to be injured, reducing patient morbidity and mortality [37]. Other forms of arterial injuries, such as pseudo-aneurysm, AV fistula, amputated/truncated vessel and intimal tear/dissection have also been described as indirect signs of recent or ongoing arterial bleeding (but not necessary bleeding during CT examination). Similarly, another indirect sign of bleeding is represented by retroperitoneal hematoma, which may be present without signs of contrast extravasation [10]. Finally, additional indications for AE include age older than 55 years [30], a systolic blood pressure < 90 mmHg [38], and a pelvic hematoma > 3.35 cm [10].

In pelvic trauma, embolization can be performed in two different ways: non-selective or selective embolization. The non-selective (proximal) embolization consists of the occlusion of the anterior and posterior division branches of both internal iliac arteries. The selective (distal) embolization is carried out by the occlusion of only the bleeding vessels. The non-selective embolization is advisable in cases of multiple bleeding vessels or in case of high suspicion of multiple arterial injuries due to multiple bone fractures in hemodynamically unstable patient. On the other hand, selective embolization is generally preferred because of its reduced risk of potential complications due to the organ ischemia, especially if subsequent surgical intervention is planned [39]. Non-selective embolization, according to some authors, is indicated to minimize the procedure time when the patient is unstable or has multiple other life-threatening injuries. However, according to other authors, selective embolization for pelvic hemorrhage is not a time-consuming procedure and can be done as rapidly as proximal embolization, without lowering the survival rate for trauma victims [40].

**Fig. 1:**
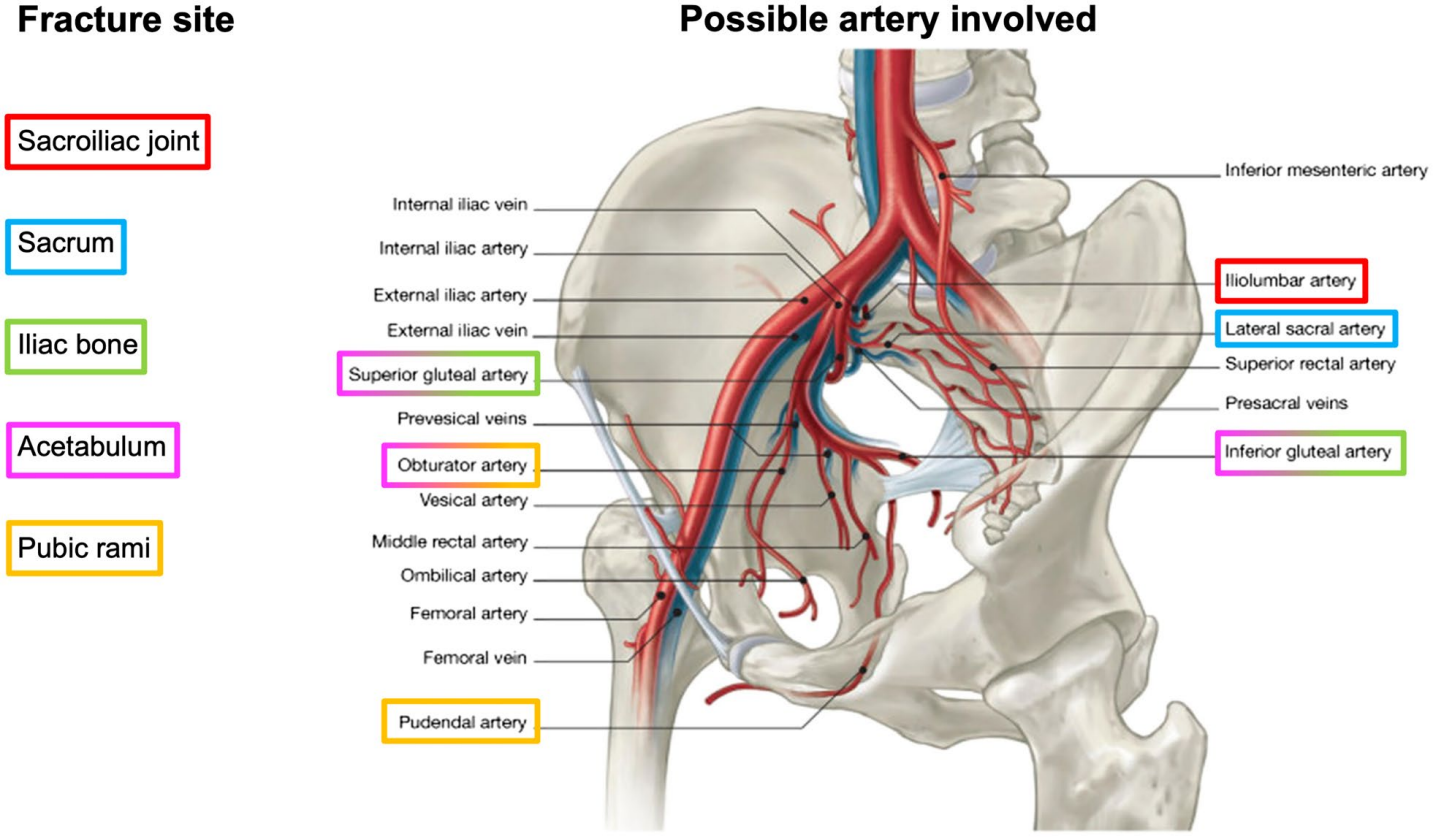
Fracture site related to the pelvic artery more likely to be injured by that kind of fracture (modified from [49], artist: Mr Philippe Payet)

Several studies reported that the location of fractures and hematomas on plain radiography or CT can be used to predict which artery has been injured and can guide selective (distal) embolization [30, 41]. Iliac fractures and/or sacro-iliac joint disruption can commonly cause injury to the iliolumbar artery while acetabular and/or pubic rami fractures can lead to obturator artery damage. The most commonly identified sources of arterial bleeding are the pudendal and the superior gluteal arteries, whereas the external iliac artery is less frequently involved [42]. Figure 1 shows a useful scheme that associates a fracture site with the pelvic vascular territory more likely to be injured by that kind of fracture. Interventional radiologists must keep in mind this scheme when treating bleeding from pelvic trauma. Furthermore, it is important to underline that pre-procedural CT evaluation can help in angiographic planning, identifying any possible anatomical variant, underlying pathologies, and or additional injuries [43].

This strategy, which contemplates the use of AE in the management of bleeding from pelvic trauma whenever contrast extravasation is detected on CT, demonstrates one important limitation: during angiographic study prior to the embolization, it may not detect the blush documented in the pre-procedural CT. The discrepancy in demonstrating active bleeding between CT and the subsequent angiographic study has been postulated to be caused by vessel spasm, which is thought to be secondary to the local inflammatory response generated by the bleeding or hypotension [44]. However, in our opinion, further studies are needed, even retrospective for obvious ethical reasons, to understand which clinical or imaging feature can correlate to a successful outcome in the absence of AE in case of these discrepancies.

### Algorithm of management of patients with pelvic fractures

According to the literature, the initial management of patients with pelvic trauma still follows the principles of the Advanced Trauma Life Support (ATLS) protocol, written in 1978 [45] and now on its tenth edition [46], based on the patient’s hemodynamic stability and response to volume resuscitation. The first maneuver, in cases of open book fractures is to apply a pelvic binder that can be quickly and easily placed directly on the accident site; however, temporary binders do not guarantee mechanical stabilization such as external fixator and they must be removed within 24 h to avoid pressure sores on the patient [17].

The most commonly used pelvic fracture classification systems that serve as a basis for therapeutic decision-making include the alpha-numeric Arbeitsgemeinschaft für Osteosyn-thesefragen/Orthopaedic Trauma Association classification, the historic classification by Marvin Tile, and the mechanistic classification by Young and Burgess [20]. However, all the above-mentioned classifications lack of a predictive correlation between the purely anatomic and mechanistic criteria and the hemodynamic response of the patients.

To overcome this limitation, the World Society of Emergency Surgery (WSES) published a new classification which is based on a combination of the anatomic/mechanistic classification by Young and Burgess in conjunction with hemodynamic stability based on ATLS guidelines [47, 48]. Based on these combined criteria, the new WSES classification stratifies pelvic trauma into three grades of severity:

- Minor injury (WSES grade 1): in case of mechanically stable injuries and a hemodynamically stable status;
- Moderate injury (WSES grade 2–3): in case of mechanically unstable injuries and a hemodynamically stable status; and
- Severe injury (WSES grade 4) in case of mechanically unstable injuries and a hemodynamically unstable status.

After the initial management according to ATLS guidelines, the patient can be categorized based on its hemodynamic response to volume resuscitation as:

- Responder: when responds adequately to fluid resuscitation and holds the systolic blood pressure > 90 mmHg.
- Transient responder: when temporarily responds to fluid resuscitation and at least transiently holds the systolic blood pressure > 90 mmHg.
- Non-responder: when the systolic blood pressure remains < 90 mmHg despite fluid resuscitation.

Hemodynamically stable patients, including “responders” and “transient responders”, are taken to the have a contrast-enhanced CT. Only patients with contrast extravasations on CT subsequently undergo AE before being transferred to intensive care unit (ICU) or OR for pelvic stabilization and PPP [48].

Hemodynamically unstable patients (“non-responder”), according to the algorithm proposed by the First Italian Consensus Conference on pelvic trauma [17], have different management strategies linked to Focused Sonography for Trauma (FAST) results. If there is the presence of free fluid in the abdomen (positive FAST), the patient will go to the OR, be urgently prepared for an exploratory laparotomy and treated with pelvic stabilization and PPP. If free fluid is absent in the abdomen (negative FAST), patients will need only a PPP and external pelvic fixation.

Until this point, the decision-making process for unstable patients due to bleeding after pelvic trauma does not involve the interventional radiological procedures. Subsequently, the therapeutic management of unstable patients essentially depends on their hemodynamic status.

In the case of persistent hemodynamic instability with ongoing bleeding, even following surgery, subsequent angiography is performed in order to identify any sign of arterial bleeding and, eventually, proceed with an embolization [17]. Then, if AE allows reaching hemodynamic stability, the patient is transferred to ICU [17]. The reason why unstable patients empirically undergo angiography, without prior CT examination, is because of sacro-iliac joint disruption and hypotension. These were identified to be independent predictors of contrast extravasation on angiography, making a CT scan unnecessary in these life-threatening cases [49].

In the case of hemodynamic stability, achieved following both surgery or external fixation/PPP, the patient is investigated with a CT scan [17]. If a contrast blush is present, this is highly predictive of arterial extravasation on angiography, therefore, the patient will undergo AE [32]. In case of negative response of the CT for blush, the patient is directly transported to ICU [17].

### How angiography could overcome algorithm’s limits

As emerged during the first Italian consensus conference [17] and discussed above, AE is considered only for stable patients with an arterial blush on CT scan and for patients who remain unstable after surgery and external fixation/PPP. However, from this point on, in the therapeutic algorithm for the management of these patients, after the word “ANGIO” [17], there are no reports on the different possibilities that an interventional radiologist has to face during the procedure. Furthermore, variations exist in the techniques and materials for performing AE in bleeding patients with pelvic trauma. Due to these differences, the outcomes differ among different published series. One of the possible reasons why this was lacking in the first Italian consensus conference was the fact that the initial algorithm drafting involved only three interventional radiologists out of 41. However, as already reported, after the early maneuvers, the interventional radiologists play a key role in the treatment of the patient with pelvic trauma, both in cases of hemodynamical stability and instability despite surgical and non-surgical procedures. Therefore, the achievement of an optimized and standardized algorithm for AE, after the word “ANGIO” [17], in the guidelines for the management of pelvic trauma is crucial to create correct and comprehensive management of these patients.

The present study aims to analyze and highlight all possible scenarios following the referral of the patient with pelvic trauma to interventional radiologists, currently not codified in published guidelines [17]. Furthermore, according to these suggestions, the present paper tries to expand the previous drafted algorithm exploring the role of the interventional radiologist, especially given the multidisciplinary setting.

First of all, regarding the angiographic procedure itself, the standard approach should start via the right common femoral artery, in order to facilitate the work of the interventional radiologist.

**Fig. 2:**
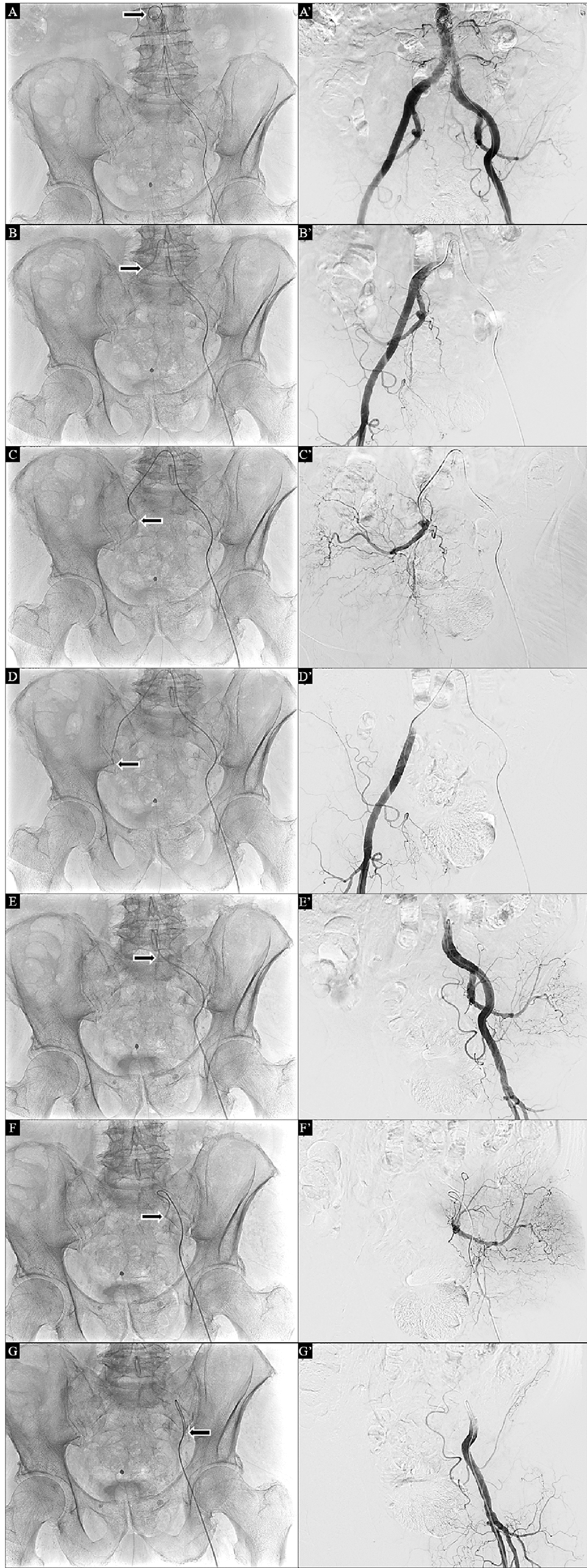
The standard approach for a correct proximal angiographic study in a bleeding pelvic trauma includes studies performed after the insertion of the catheter in the distal abdominal aorta (**A**), the right common iliac artery (**B**), the right internal iliac artery (**C**), the right common femoral artery (**D**), the left common iliac artery (**E**), the left internal iliac artery (**F**), and the left common femoral artery (**G**). The subsequent digital subtracted images of these studies are shown in the corresponding panels (**A**′–**G**′)

Secondly, it is important to standardize the terminology. Proximal angiography is considered a study conducted with the tip of the catheters in the distal abdominal aorta, in left and right common iliac arteries, left and right internal iliac arteries, and left and right common femoral arteries (Fig. 2). Selective studies are considered those in which are selectively catheterized arteries located distally with respect to the previously mentioned vessels catheterized for proximal studies.

Many embolizing materials are available and the choice of which type to use depends on interventional radiologist’s experience and, especially, may vary due to the different outcomes: an absorbable embolizing material (i.e., gelatin sponge) is preferred when the bleeding site affects the vital organs, such as penis, vagina, intestine, marrow, testes, uterus and ovaries; on the contrary, a non-absorbable embolizing material (i.e., glue) is preferable in terminal vessels of non-vital organs, such as muscles [50].

**Fig. 3:**
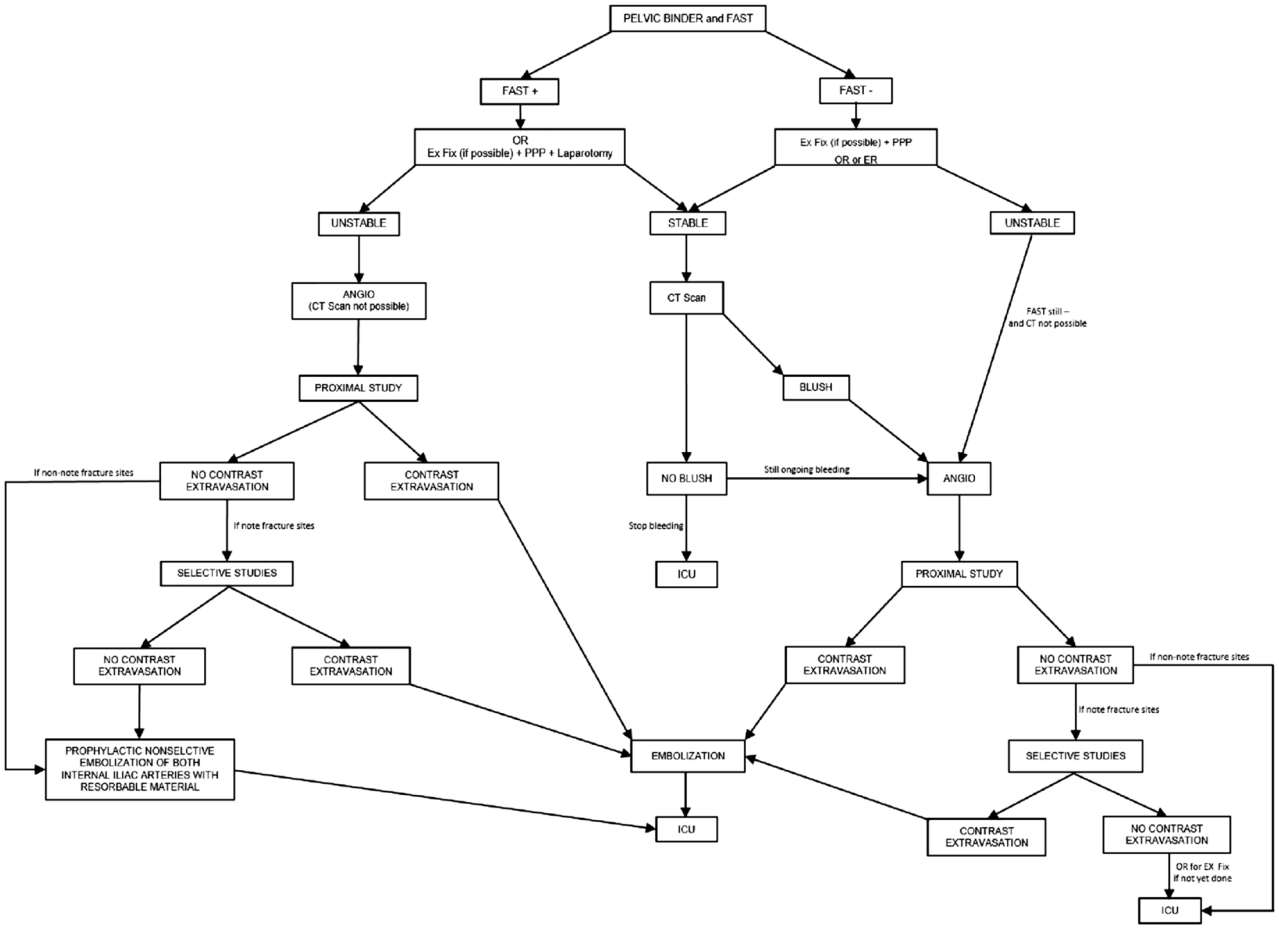
Treatment algorithm for pelvic trauma (modified from [14])

The possible scenarios for the interventional radiologist after the word “ANGIO” actually non-standardized in the treatment algorithm of pelvic trauma^17^ are the following (summarized in Fig. 3).

### Scenarios for stable patients (Sp)

**Fig. 4:**
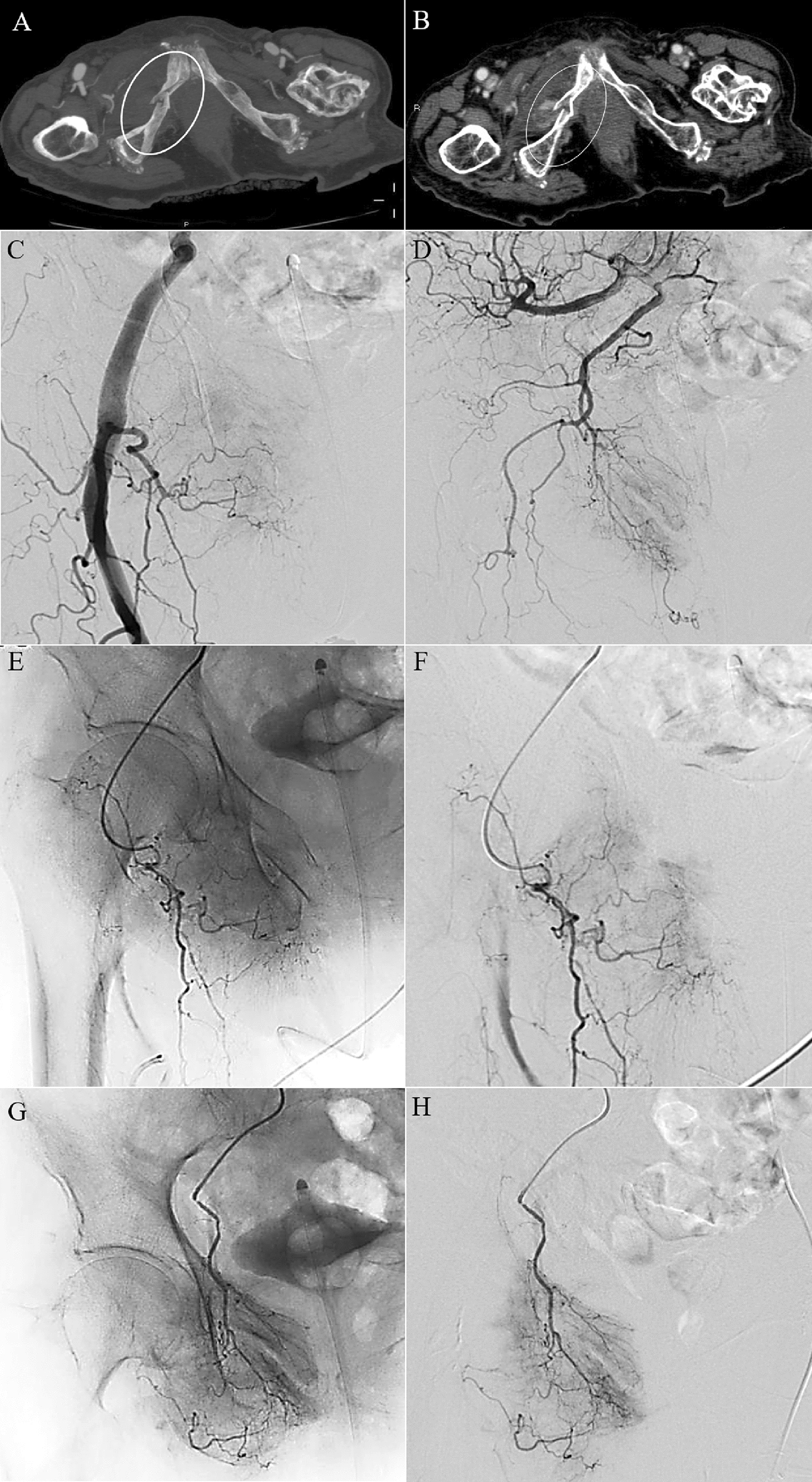
Axial CT of a stable patient with a fracture of the right pubic ramus (circled in **A**) presenting with a small focal region of contrast extravasation (circled in **B**). Proximal angiograms, from the right external iliac artery (**C**) and from right internal iliac artery (**D**), showing no signs of contrast extravasation in correspondence of the site of the findings on CT. The selective catheterizations of the suspected injured arteries based on CT findings, for the digital studies, i.e., of the right external obturator artery [non-subtracted (**E**) and subtracted (**F**) angiograms] and of the right internal obturator artery [non-subtracted (**G**) and subtracted (**H**) angiograms], showing no signs of bleeding. According to the proposed algorithm, the angiographic study was considered completed and the empiric embolization was not performed

**Fig. 5:**
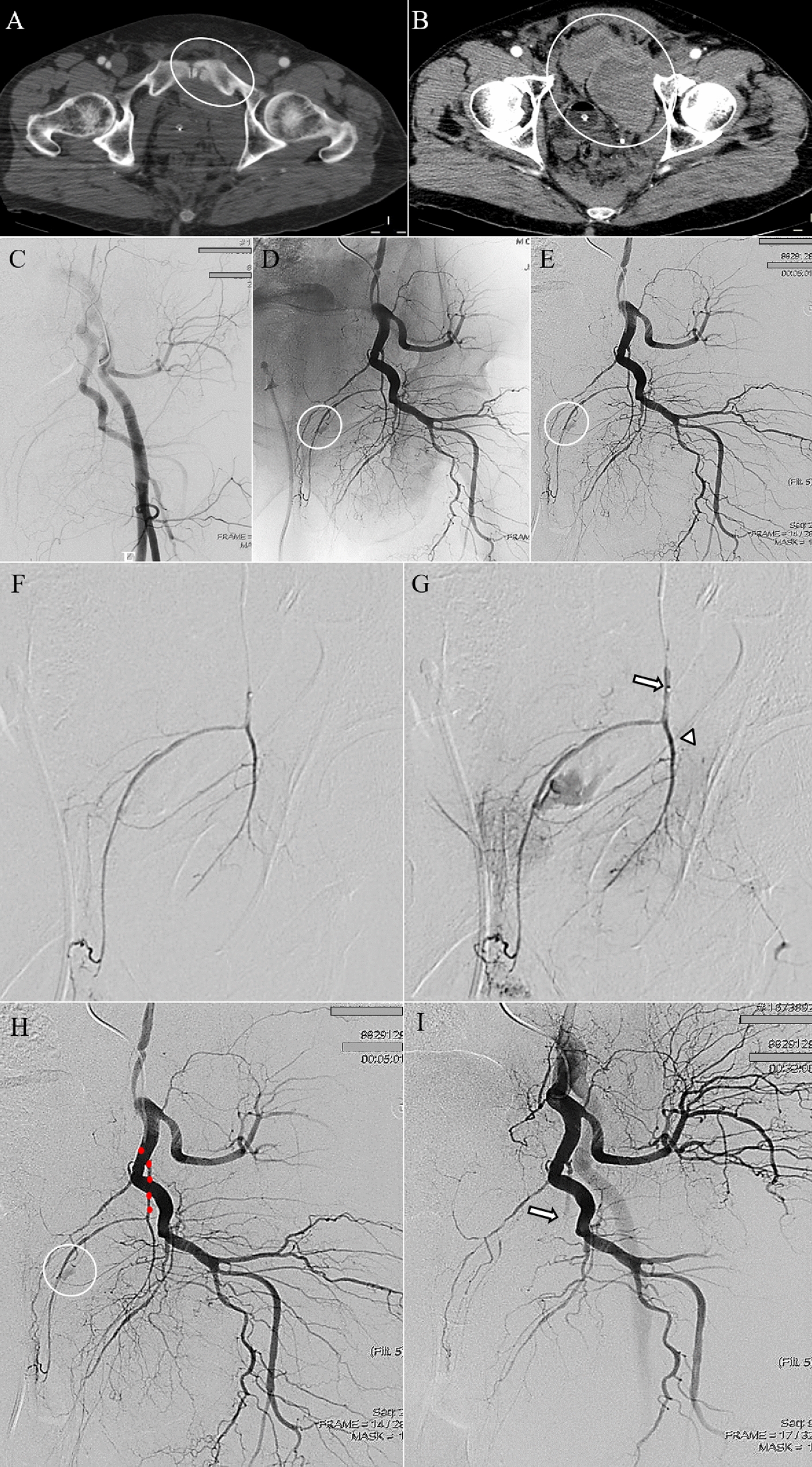
Axial CT of a stable patient with a fracture of the left pubic ramus (circled in **A**) and pelvic hematoma (circled in **B**). Proximal non-subtracted angiogram of the left external iliac artery (**C**) showing no focal “blush”. The subsequent selective study of the left internal obturator artery demonstrating the contrast blush [circled in subtracted image (**D**)] in correspondence consistent with injury lesions on CT and suitable for superselective embolization; digital subtracted image of the same patient, allowing a more accurate depiction of the arterial extravasation (circled in **E**). Selective catheterization of the injured artery (**F**). The arterial branch responsible for the blush was embolized with Glue, a definitive embolic agent (head arrow in **G**) while the upper obturator branch that supplies external genitalia is closed with Spongel, an absorbable embolic material (arrow in **G**). Compared to the pre-procedural angiography in which are highlighted the vessel (red points in **H**) responsible for bleeding (circled in **H**), the post-embolization angiogram confirms a successful occlusion only of the injured artery (arrow in **I**) with the regular patency of the remaining vessels

1. Stable patients with a blush on CT plus contrast extravasation on angiography (Sp/CT+/A+): guided by CT positivity for arterial bleeding, direct catheterization of the detected injured artery is needed and embolization should be performed.
2. Stable patients with a blush on CT and no contrast extravasation on angiography (Sp/CT+/A−): despite the detection of blush on CT, proximal angiography may be negative due to vasospasm [58] or due to the possibility of spontaneous resolution in the time elapsed between CT and angiogram [34], placing an incredible diagnostic dilemma: when should the angiographic study be considered completed and, therefore, concluded? The search for the bleeding site can be considered concluded only after following the previously stated recommendations, i.e., after performing a direct catheterization of the suspected artery with a blush on CT and of the principal pelvic arterial branches possibly involved by the fractures documented on CT according to the scheme in Fig. 1. Only after selective catheterization of the possible involved arteries demonstrated no direct or indirect signs of bleeding, the angiographic study could be considered complete (Fig. 4). If, after selective catheterizations, the source of bleeding is detected, the scenario becomes the same as the previous statement (#1).
3. Stable patients with negative CT but with ongoing bleeding plus contrast extravasation on angiography (Sp/CT−/A+): a negative CT could simply indicate that it is not possible to identify the source of bleeding with this imaging examination at this time. However, the patient could still have ongoing bleeding. Therefore, in this scenario, after a negative proximal study, selective catheterizations of the arterial branches more frequently involved according to the fracture’s site (see Fig. 1) are needed. Once the bleeding site is identified, the injured artery will be embolized (Fig. 5).
4. Stable patients with negative CT but with ongoing bleeding and no contrast extravasation on angiography (Sp/CT−/A−): as in the previous scenario (#3), the search for the bleeding site can be considered completed and the angiographic study concluded (without AE) only after proximal and distal catheterizations of the arterial branches more frequently involved according to the fracture’s site demonstrated on imaging such as radiography (see Fig. 1) do not demonstrate any contrast extravasations.

### Scenarios for unstable patients (Up)

In these scenarios, damage control techniques, such as PPP, damage control laparotomy and REBOA are the preferred strategies. AE in unstable patients can be considered in a hybrid OR, as a part of multidisciplinary interventions, and are performed after damage control procedures, as a completion of the hemostasis. The scenarios for unstable patients are the following:

5. Unstable patients, not investigated with CT, with negative FAST plus contrast extravasation on angiography (Up/noCT/FAST−/A+): since CT cannot be performed, angiography is the only option to identify a possible suspicious bleeding site. Therefore, it is mandatory to perform a proximal study, as described above. In case of a negative proximal study, in the availability of previous imaging such as radiogram that can be used as a guide to identifying the most likely injured arteries according to the fracture site, it is mandatory to perform selective studies, embolizing eventual sources of bleeding.
6. Unstable patients, not investigated with CT, with negative FAST and no contrast extravasation on angiography (Up/noCT/FAST−/A−): as in the previous scenario (#5), the search for the bleeding site can be considered completed and the angiographic study concluded (without AE) only after proximal study and distal catheterizations of the arterial branches more frequently involved according to the fracture’s site demonstrated on imaging such as radiography (see Fig. 1) do not demonstrate any contrast extravasations.
7. Unstable patients, not investigated with CT, with positive FAST plus contrast extravasation on angiography (Up/noCT/FAST+/A+): the interventional radiologist could apply the same strategy of scenario #5.
8. Unstable patients, not investigated with CT, with positive FAST and no contrast extravasation on angiography (Up/noCT/FAST+/A−): the interventional radiologist could apply the same strategy as in scenario #6 with the unique difference that, if neither proximal nor selective studies have shown any signs of bleeding, after a rapid agreement with the anesthetist, it is highly recommendable to perform a prophylactic non-selective bilateral internal iliac artery embolization with resorbable materials (such as Gelfoam) for temporary occlusion, even more given the persistent hemodynamic instability and positive FAST result. This theory is supported by the evidence that bilateral internal iliac embolization in case of a negative angiography may aid in hemorrhage control for those patients still being actively transfused as evidenced by the decreased amount of blood products infused [51–55]. Moreover, no difference in survival, ICU/hospital length of stay, complications were found between embolized patients and those that received no intervention when the angiogram was negative [56].

All the above considerations must take into account the possible disadvantages in the transportation of a severely injured patient to the angiographic room [57]. However, the increasingly widespread use of hybrid OR, where trauma surgeons, orthopedic surgeons, and interventional radiologists can work together, is progressively enabling the simultaneous or alternate performance of the initial assessment, resuscitation, damage control surgery, and endovascular procedures, without the need to transfer the patient to the radiological room or the OR [9].

Moreover, although AE is considered to be a safe technique, there are reports in the literature of complications, such as gluteal and femoral head necrosis, thigh or buttock claudication, bladder necrosis, lower extremities paresis, and sexual dysfunction. However, controversy exists on whether these complications are due to embolization or the trauma itself [37]. Regardless of the cause, the physician must be vigilant in the detection of complications because they affect outcomes and can complicate surgical management. Despite non-selective embolization of the internal iliac artery, especially bilateral embolization, has been suggested to have higher complication rates than selective embolization, several studies reported no complications overall or no complications associated with the bilateral non-selective procedure [39].

Furthermore, interventional radiologists are not available at all times in most hospitals and often serve on-call [58, 59].

## Conclusions

In conclusion, over the years the role of interventional radiology in the management of patient bleeding due to pelvic trauma has evolved and increased. The current guidelines on the management of these patients do not adequately reflect or address the varied nature of injuries faced by the interventional radiologist. It seems that the result of accessing the angiographic room is uniform but, unfortunately, this is not the case, because angiographic treatments have several facets. This would also explain, at least in part, the different results in the outcomes of the different series published in the literature. Therefore, standardization of each step concerning the angiographic procedure in the patient bleeding due to pelvic trauma is necessary. This study reviews the current literature and critically proposes a possible standardization of the angiographic procedure in this setting, both in relation to how to perform the examination, both in terms of the technique and embolization materials, and in relation to the common terminology to be used. Further exploration and studies, possibly through expert consensus, are required in this topic to obtain data and consensus on management strategies.

## Data Availability

Not applicable.

## Abbreviations

AE: Angioembolization
PPP: Preperitoneal pelvic packing
REBOA: Resuscitative endovascular balloon occlusion of the aorta
CT: Computed tomography
OR: Operating room
ICU: Intensive care unit
FAST: Focused sonography for trauma
Sp: Stable patient
Up: Unstable patient
